# Mechanical Anisotropy and Fatigue Behavior of 3D-Printed Dentures: A Comparison with CAD/CAM Milled Bases After Thermomechanical Aging

**DOI:** 10.3390/jfb17060297

**Published:** 2026-06-15

**Authors:** Mohamed Ahmed Alkhodary, Ramy Elmoazen, Bandar Awadh Alresheedi, Ali Alenezi, Naji Alharethi, Rawan Alrethia

**Affiliations:** 1 Department of Prosthetic Dental Sciences, College of Dentistry, Qassim University, Saudi Arabia; b.alresheedi@qu.edu.sa (B.A.A.); ali.alenezi@qu.edu.sa (A.A.); n.alharthi@qu.edu.sa (N.A.); ra.alrethia@qu.edu.sa (R.A.); 2 School of Computing, University of Eastern Finland, Yliopistokatu 2, 80100 Joensuu, Finland; ramy.elmoazen@uef.fi

**Keywords:** 3D printing, dental materials, laboratory technique, flexural strength, fatigue resistance, CAD/CAM milled bases, after artificial aging, chewing simulation

## Abstract

To investigate the effect of print orientation (0°, 45°, and 90°) and artificial aging on flexural strength and fatigue resistance of 3D-printed denture bases compared to CAD/CAM milled controls, we fabricated 320 maxillary complete dentures, divided into 8 groups based on the fabrication method: horizontal, oblique, and vertical printing, alongside milled controls. Half of the specimens in each group were pre-conditioned via thermocycling and 240,000 cycles of chewing simulation. All specimens underwent static flexural strength testing and cyclic fatigue testing, followed by SEM fractography. The CAD/CAM milled bases demonstrated the highest mechanical durability, with non-aged specimens peaking at 149.43 ± 5.35 MPa. The horizontally 3D-printed non-aged specimens yielded the highest flexural strength (101.14 ± 4.80 MPa), while vertically printed aged specimens recorded the lowest (70.35 ± 8.18 MPa). Artificial aging degraded flexural strength uniformly across all orientations. Conversely, cyclic loading disproportionately devastated the older people’s vertical group, resulting in a 70% fracture rate. Fractography corroborated these findings, revealing severe interlaminar delamination in vertical builds, contrasting with cohesive, trans-layer fractures in horizontal prints. In conclusion, Horizontal orientation provided improved structural durability; however, CAD/CAM milled dentures remain superior and are recommended for long-term clinical applications.

## 1. Introduction

While CAD/CAM-milled polymethyl methacrylate (PMMA) blocks are produced under highly controlled industrial polymerization conditions, resulting in dense, homogeneous structures with superior mechanical performance, 3D printing reduces material waste and customizes streamlined workflows [1,2,3,4,5,6]. Nevertheless, a recurring concern across literature is the relatively inferior or inconsistent mechanical behavior of 3D-printed denture base resins compared with their milled counterparts, particularly in flexural and fatigue fracture resistance [7,8,9,10,11,12,13,14,15].

A critical factor that distinguishes 3D printing from milling is the successive layer deposition process, which leads to material anisotropy. Among the various printing parameters, build orientation (printing angle) has been consistently identified as a key determinant influencing the structural integrity, accuracy, and surface characteristics of printed denture bases. Alterations in printing angle affect polymerization dynamics and collectively govern the final mechanical properties [16,17,18,19,20,21,22,23,24,25,26,27].

Several investigations have reported that horizontally oriented specimens (0°) exhibit superior flexural strength, likely due to favorable alignment of layers relative to applied stresses [28,29,30,31,32,33]. Conversely, vertically oriented specimens (90°) have often demonstrated reduced strength and increased susceptibility to fracture, attributed to weaker interlayer adhesion [34,35,36,37,38,39]. Intermediate orientations, particularly 45°, have been suggested to provide a more balanced stress distribution and, in some cases, improved overall performance [7,9,12,13]. Despite these trends, inconsistencies persist, with some studies emphasizing the superiority of specific angles depending on the printing technology, resin type, or testing conditions [14,15,16,17,40,41,42,43,44].

In addition to mechanical properties, printing orientation has also been shown to significantly influence surface roughness, dimensional accuracy, and microbial adhesion. Surface topography appears to be more sensitive to building angles than to layer thickness [8,9]. When compared to CAD/CAM milling, the limitations of 3D printing become more evident and suggest the need for optimization of printing parameters, particularly build orientation, which is essential to bridge this performance gap [18,19,20,42,43,44].

Despite the growing body of evidence, there is no clear consensus regarding the optimal printing angle for denture base fabrication. The variability in studies design, used materials, testing methodologies, and the ongoing discrepancies in the literature regarding the effects of thermocycling and chewing simulation on 3D-printed denture bases, this study aimed to investigate the of printing orientations of horizontally (0°), obliquely (45°), vertically (90°) nested denture bases on their flexural strength, fatigue resistance, and the pattern of their fracture surfaces. The null hypothesis was that neither printing orientation nor thermomechanical aging would significantly affect the flexural strength, fatigue resistance, or fracture characteristics of 3D-printed denture bases, and that no differences would exist between 3D-printed and CAD/CAM milled denture bases.

## 2. Materials and Methods

### 2.1. Study Design

This controlled laboratory experimental study evaluated the effect of printing orientations, horizontal (0°), oblique (45°), and vertical (90°), on the flexural strength and fatigue resistance of maxillary complete dentures, before and after thermocycling aging and chewing simulation. CAD/CAM milled maxillary denture bases were used as controls.

A total of 320 dentures were allocated into eight experimental groups according to fabrication methods (horizontal, oblique, vertical 3D printing, or CAD/CAM milling) and aging condition (aged or non-aged). Each group contained 40 specimens, equally divided between flexural strength testing and fatigue testing (Table 1).

Sample size calculation was conducted using power analysis. Based on a pilot study (effect size f = 0.45), with α = 0.05 and Power (1 − β) = 0.80, the minimum required sample size per subgroup was 18 dentures. To compensate for potential specimen loss, 20 dentures per subgroup were fabricated.

### 2.2. Sample Preparation

**Figure 1:**
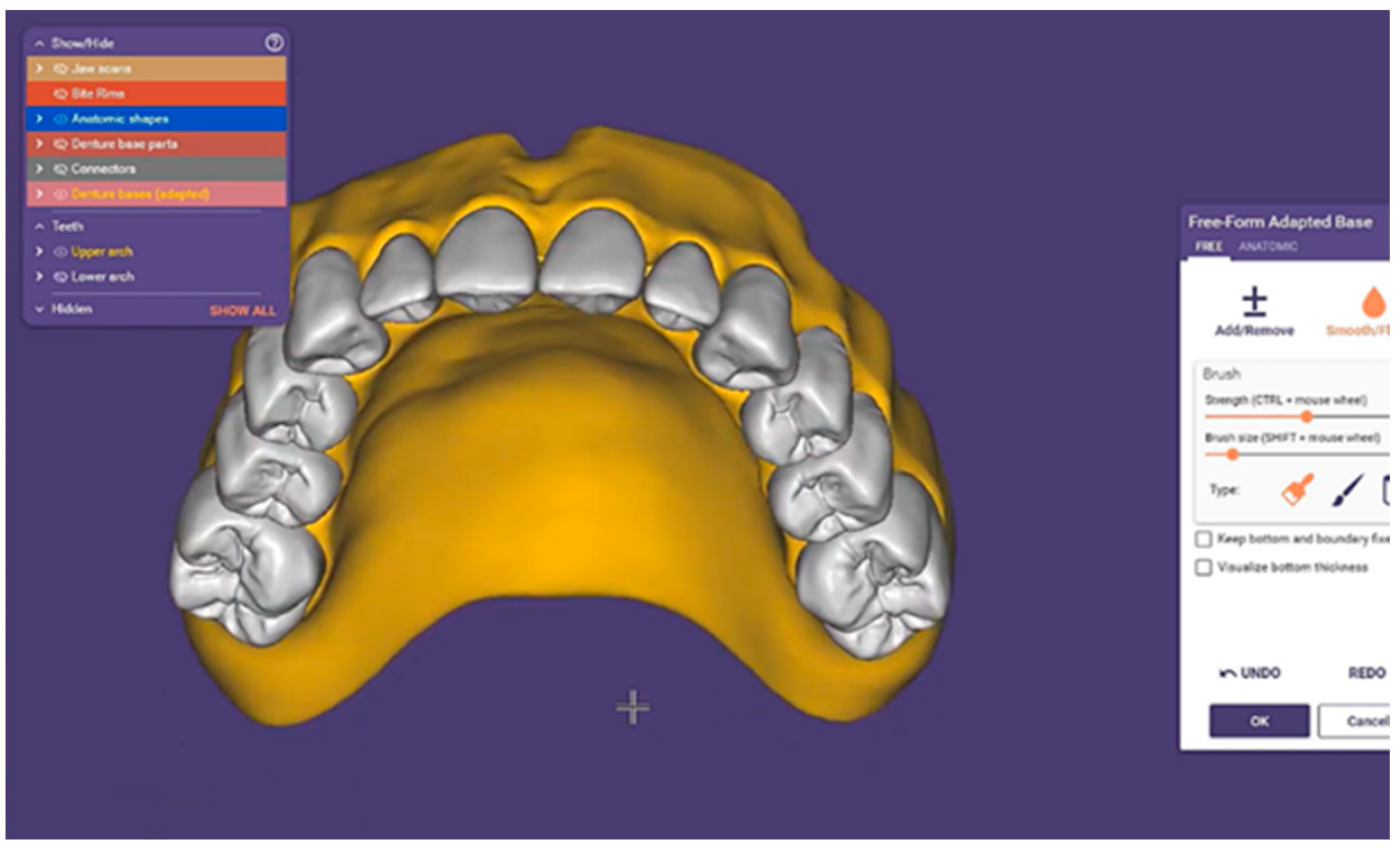
CAD design of a maxillary complete denture sample.

**Figure 2:**
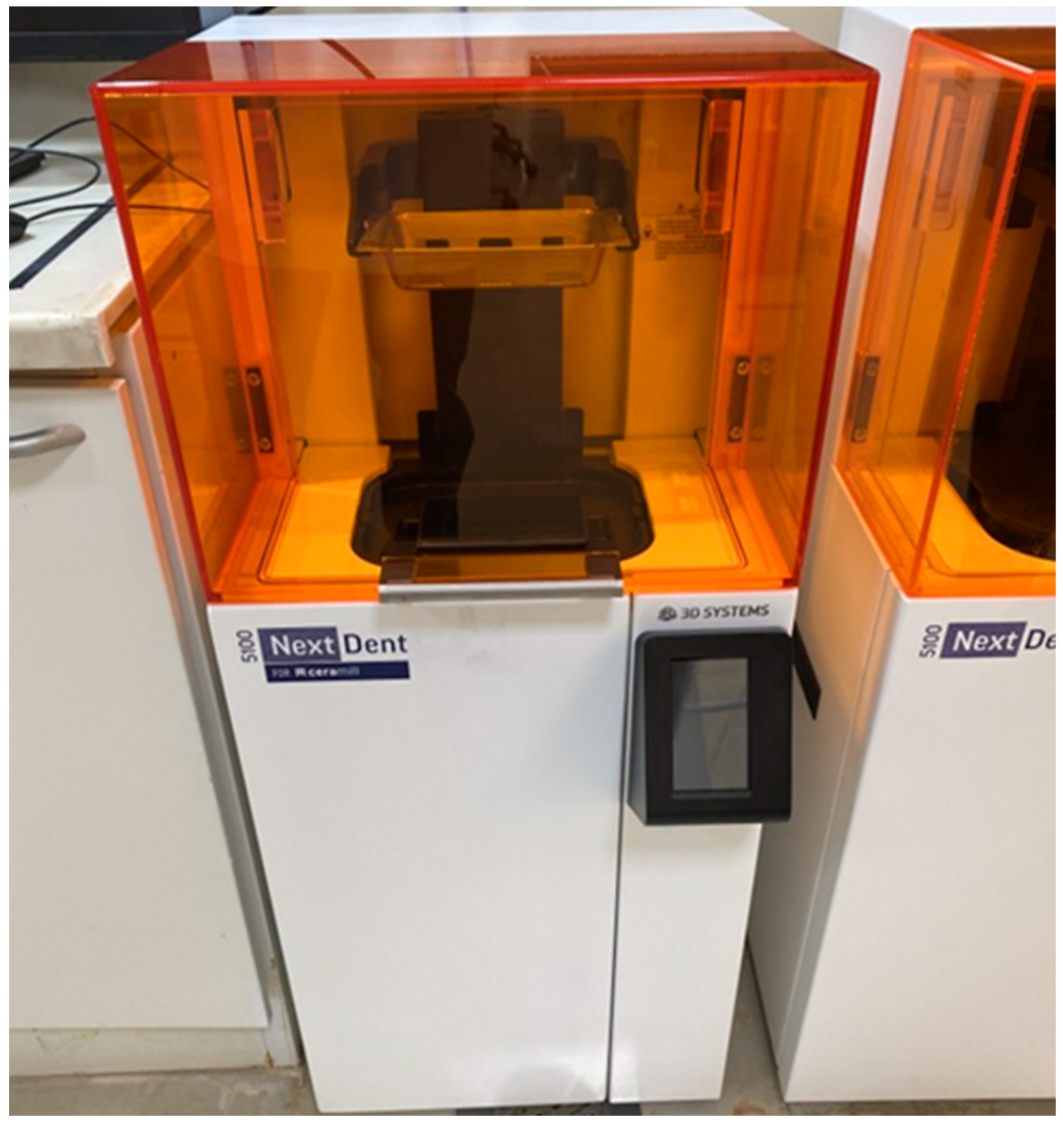
Next Dent 5100 3D printer.

**Figure 3:**
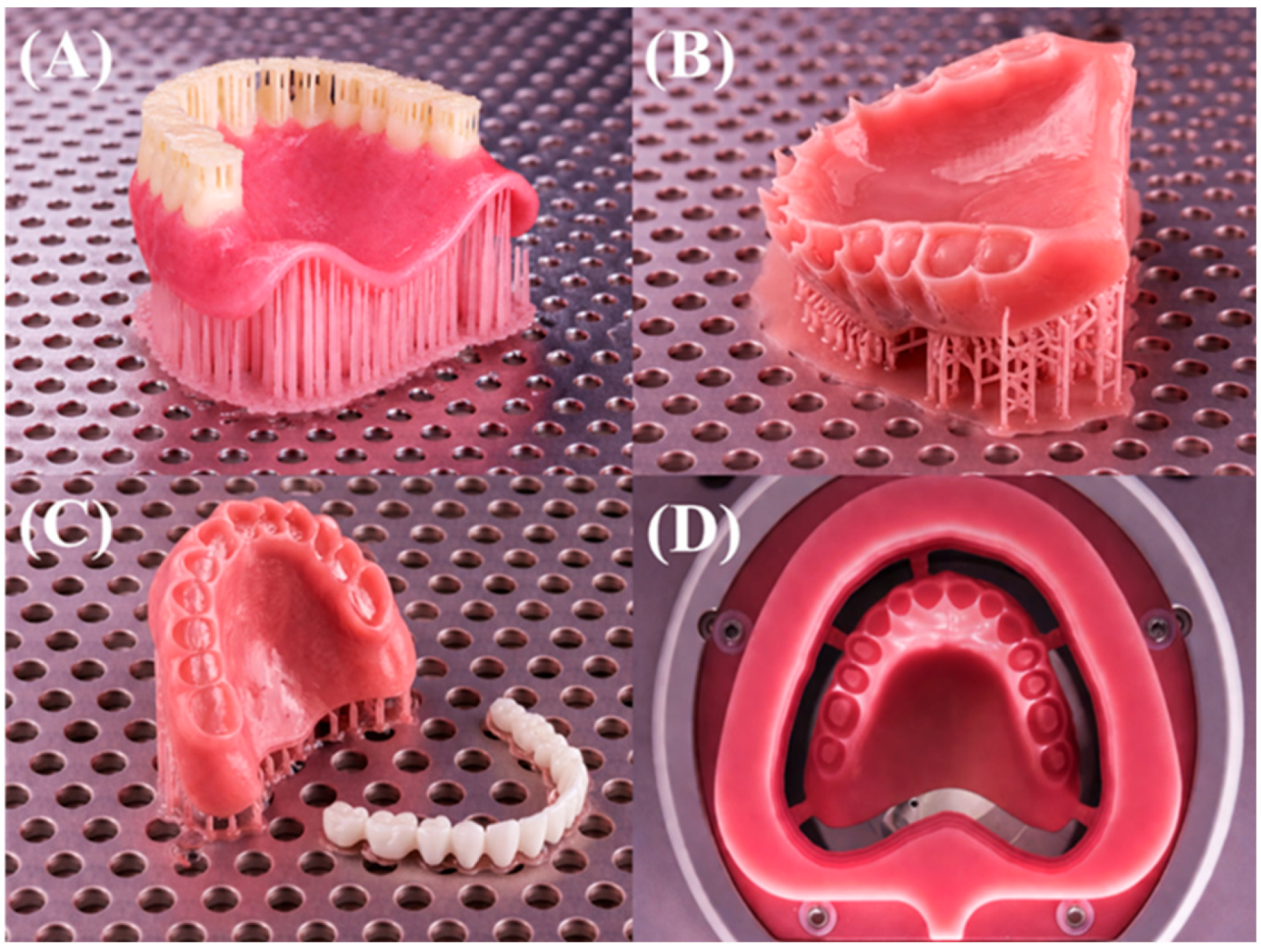
(**A**) Horizontally printed denture base, with the 3D printed teeth seated in place before removing the supporting pillars; (**B**) 45° printed denture base; (**C**) Vertically printed denture bases; (**D**) CAD/CAM milled denture base.

A standardized edentulous maxillary master model was cast using a high-strength epoxy resin, scanned with bench top scanner (Kavo ARCTICA AutoScan, KaVo Dental GmbH, Biberach an der Riß, Germany), and its CAD file was imported to Exocad 3.1 to design the dentures for all groups (Figure 1), then the denture bases and teeth were printed using Next Dent 5100 3D printer (NextDent B.V., Soesterberg, The Netherlands) (Figure 2), with Next Dent Denture 3D+ resin, and Next Dent C&B micro filled hybrid for the denture teeth, at a layer thickness of 50 µm, and a build angle of 0° (horizontal) for groups I and II, 45° (oblique) for groups III and IV, and 90° (vertical) for groups V and VI (Figure 3). The same standardized edentulous maxillary master model CAD file was used to mill the denture bases and teeth for groups VII and VIII using a CAD/CAM milling machine (Dentsply Sirona, Bensheim, Germany).

**Figure 4:**
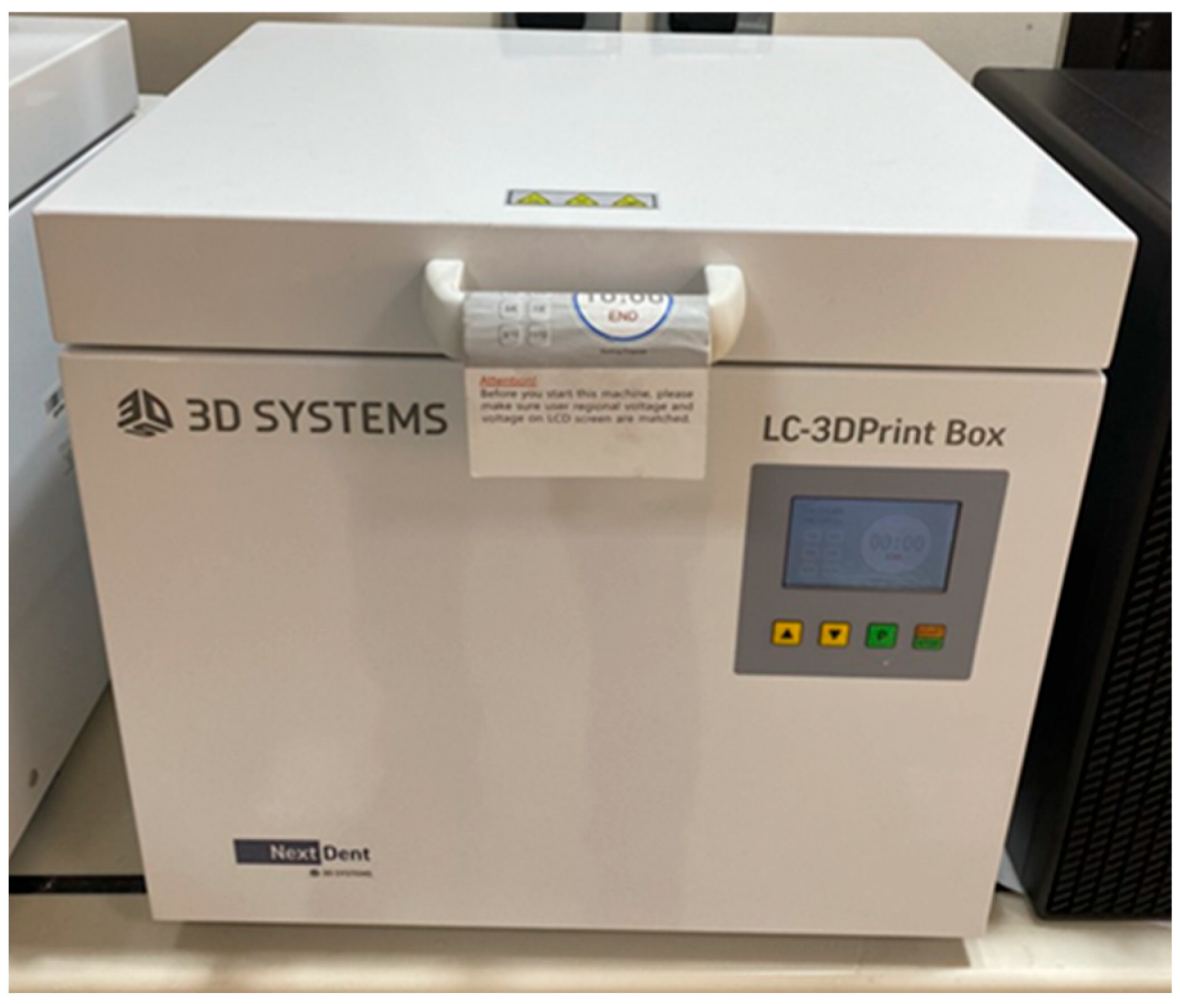
LC-3DPrint Box.

For groups I to VIII, supporting pillars were auto-generated and removed after printing. Post-curing was performed according to the manufacturer’s recommendations to standardize polymerization conditions across all specimens. Post-processing of the 3D-printed denture bases was done by 2 cycles of cleaning immersion in 99% isopropyl alcohol for 5 min, followed by air drying for 10 min. Post-printing curing was done using the LC-3DPrint Box for 30 min, during which intaglio and cameo surfaces were exposed to 15 min of UV curing at 405 nm wavelength (Figure 4). On the other hand, CAD/CAM milled parts did not need any post-milling treatment.

The denture bases of all groups were then finished and polished using successively smaller grit silicon carbide abrasive papers in the presence of water coolant. To ensure that the finishing and polishing procedures did not affect the denture bases’ thickness, dimensional verification was done using a digital caliper. The tooth sockets and the cervical areas of the teeth were sandblasted, cleaned with isopropyl alcohol, and air-dried, then a thin, uniform layer of light-curing bonding agent (Lucitone Digital Fuse) was applied to both the sockets and the teeth. Once the teeth were firmly seated in place, a handheld UV light was used for approximately 10–20 s per tooth to stabilize the position. The final post-processing curing was done in the LC-3DPrint Box at 60 °C for 10 to 30 min.

### 2.3. Thermomechanical Aging (Pre-Conditioning)

**Figure 5:**
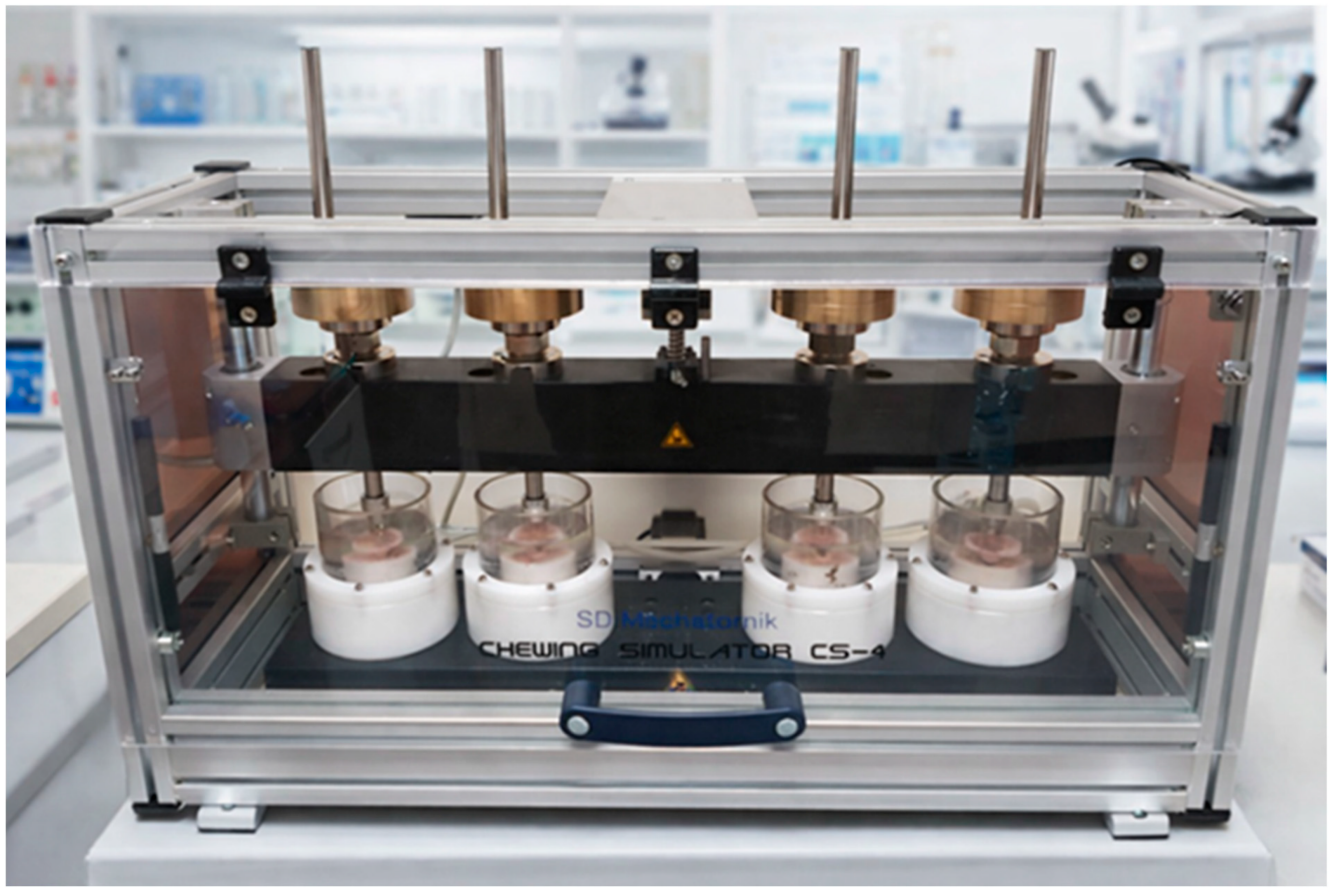
Samples subjected to chewing simulation and thermal aging in the Chewing simulator (CS-4.4, SD Mechatronik).

Chewing simulation and thermal aging were conducted for groups II, IV, VI and VIII denture bases in a chewing simulator (SD Mechatronik, Feldkirchen-Westerham, Germany) that could replicate both dynamic occlusal loading and thermal aging. One hundred and sixty replicas of the standardized edentulous maxillary master model, which was scanned for the design and fabrication of the dentures, were cast using a high-strength epoxy resin. The 120 printed dentures of groups II, IV and VI, and the 40 milled bases of group VIII were cemented onto the epoxy models using a 2 mm thick layer of polyvinyl siloxane soft relining material (Mucopren Soft, Kettenbach, Germany) with a Shore A hardness of approximately 33. This specific hardness and thickness were selected to accurately simulate the viscoelasticity and compressive resilience of the human maxillary mucoperiosteum, ensuring bio-realistic stress distribution under the denture base during cyclic loading. The denture models’ assemblies were secured into the lower sample holders of the CS-4.4 chambers using auto-polymerizing acrylic resin, with their occlusal planes oriented perpendicular to the vertical (*Z*-axis) loading bar. A standardized mandibular antagonist arch was milled from a highly cross-linked PMMA for full-arch loading of the dentures. The CS-4.4 was programmed for a dual-axis movement using a vertical load of 50 N, horizontal slide of 0.5 to 1.5 mm, descending speed of 40 mm/s, frequency of 1.2 Hz for 240,000 cycles, which represents approximately a 1-year chewing simulation (2, 6, 19). For thermocycling aging, the samples were submerged in the distilled water-filled CS-4.4 chamber, with the thermal cycle set at 5 °C ± 1 °C for the cold bath and 55 °C ± 1 °C for the hot bath, a dwell time of 60 s, and a drain/transfer time of 10 s (Figure 5). A total of 10,000 thermocycles were performed, corresponding to approximately one year of clinical service, and synchronized with the chewing simulation cycles. The thermocycling range (5–55 °C) was selected in accordance with widely accepted ISO-based dental material testing protocols (ISO 20795-1:2013 [45]), representing extreme intraoral conditions during ingestion of cold and hot substances, thereby providing a standardized and reproducible aging model. On the other hand, all non-aged control groups (Groups I, III, V, and VII) were stored in distilled water at 37 °C for a duration matching the thermomechanical aging protocol applied to the other groups [6,10].

### 2.4. Flexural Strength Testing (Static Loading)

**Figure 6:**
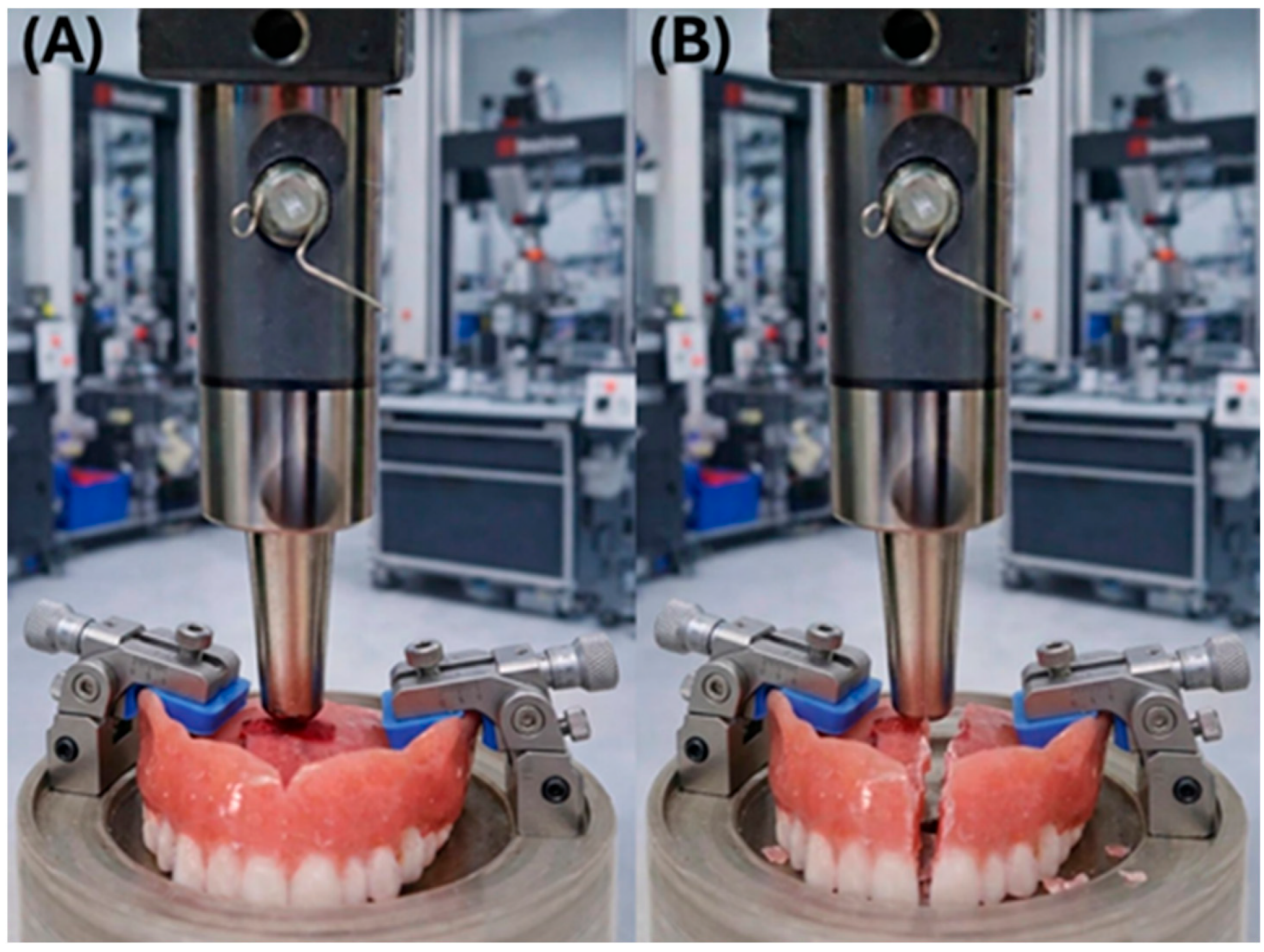
Flexural strength testing using an Instron universal testing machine: (**A**) Maxillary denture being tested for flexural strength testing until failure as seen in (**B**).

Testing the Flexural strength of dentures of all groups was then done using a universal testing machine (Instron Corp., Norwood, MA, USA) with a 5 kN Load cell, a crosshead speed of 5 mm/min, with the load applied at the center of the palate until fracture. Results were expressed in MPa (Figure 6). Flexural strength (MPa) was calculated by normalizing the fracture load to an equivalent stress value based on the denture’s effective cross-sectional geometry at the palatal midline, which represents the region of maximum tensile stress. Due to the complex geometry of complete dentures, this approach follows previously published methodologies [1,2,6,10], allowing standardized comparison across groups.

### 2.5. Accelerated Fatigue-to-Failure Testing (Cyclic Loading)

**Figure 7:**
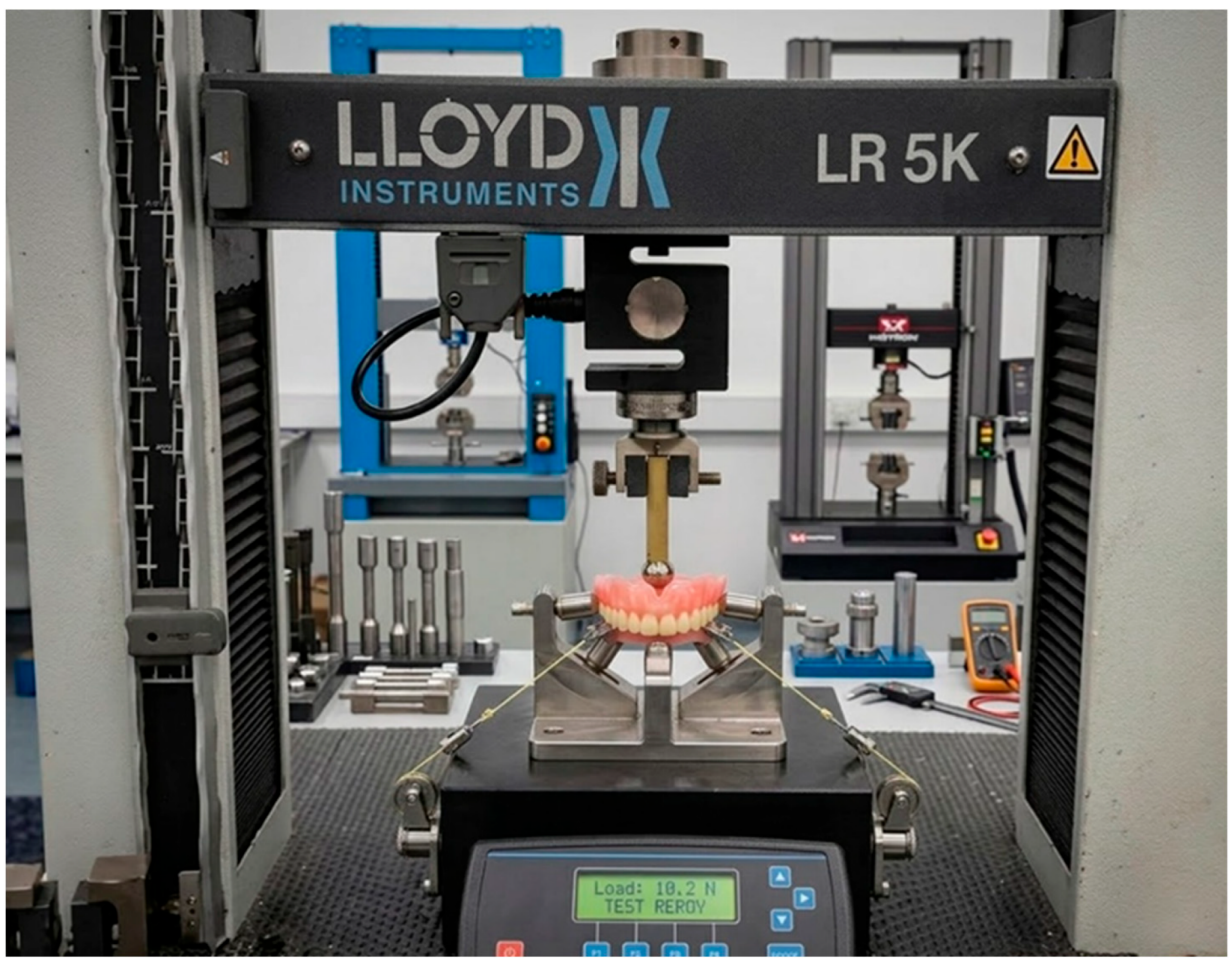
Fatigue testing using an Instron universal testing machine, with a precision-engineered stabilization system: custom-machined metal clamps grip the base of the denture, and two fine, tensioned Kevlar cables run from these clamps down to dedicated anchoring points on the black base block.

The CS-4.4 chewing simulator was utilized strictly to precondition the specimens through thermomechanical aging, simulating one year of intraoral hydrolytic and subcritical mechanical degradation. Following this aging phase, the specimens were subjected to an accelerated, high-load fatigue test in a universal testing machine to determine their ultimate cycles to failure [2,6,10]. Fatigue resistance of 20 denture bases, in each of the eight groups, was evaluated using cyclic loading in a universal testing machine (LLOYD, LR5K Plus Materials Testing Machine, Lloyd Instruments Ltd., Fareham, UK). Each denture specimen was positioned on the testing platform with the palatal midline aligned beneath a hemispherical stainless-steel indenter to reproduce the loading configuration used during flexural testing. Cyclic compressive loading was applied at the center of the palatal region using a sinusoidal waveform with a load range between 10 N and 100 N at a frequency of 2 Hz. The test was conducted for a maximum of 100,000 cycles or until fracture occurred, whichever came first. The number of cycles to failure was recorded for each specimen as an indicator of fatigue resistance. Specimens that completed the full loading cycle without fracture were considered runouts (Figure 7).

### 2.6. Fractography

Flexural strength and fatigue resistance testing of fractured samples from all groups were examined using scanning electron microscopy (JEOL Ltd., Tokyo, Japan) at ×1000 magnification to evaluate fracture features, interlayer separation, and crack propagation pathways. Samples from each group were ultrasonically cleaned in distilled water for 5–10 min to remove debris, then dried in a desiccator for at least 24 h to eliminate moisture. The samples were mounted on aluminum SEM stubs using double-sided conductive carbon tape, with the fracture surface oriented upward and centrally aligned. The mounted samples were placed in a sputter coater, and a thin, uniform conductive coating of gold (Au) was applied to improve surface conductivity. A gold coating of ~10–20 nm thickness was applied using 10–20 mA current for 120 s. The SEM Examination was conducted at ×1000 magnification under high vacuum mode (typically ~10^−5^ to 10^−6^ Torr), at 12.3 mm working distance, and an accelerating voltage of 15 kV.

### 2.7. Statistical Analysis

Descriptive statistics were computed for each group, including total sample size, number of fractured and runout specimens, and fracture rate (%). Normality was evaluated using the Shapiro–Wilk test, and homogeneity of variance was assessed using Levene’s test to validate the use of standard parametric two-way ANOVA. Aligned Rank Transform (ART) ANOVA was performed as a non-parametric alternative, using the ARTool package version 0.11.2 in R. The ART procedure aligns the response variable by removing the estimated effects of all other model terms prior to ranking. Effect sizes were reported as partial eta-squared (η^2^p), calculated from the ART ANOVA. Effect size benchmarks followed Cohen’s guidelines: small (η^2^p = 0.01), medium (η^2^p = 0.06), and large (η^2^p ≥ 0.14).

Post hoc pairwise contrasts were conducted using the ART.CON function within ARTool. Tukey’s Honestly Significant Difference (HSD) method was applied to control the orientation-level comparisons. For the Orientation × Conditioning interaction, Bonferroni correction was applied across all pairwise comparisons among the eight group combinations. All tests were applied with a significance level of *p* < 0.05. Statistical analyses were performed using R programming.

## 3. Results

### 3.1. Flexural Strength

**Figure 8:**
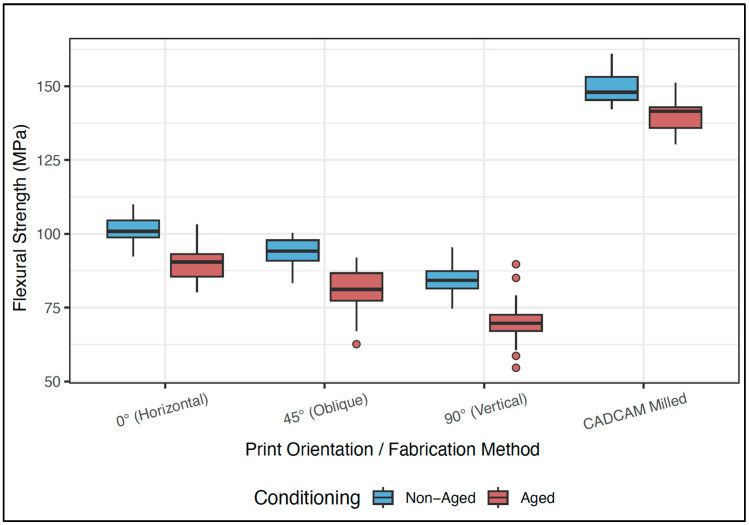
Box plot of flexural strength (MPa) across all experimental groups stratified by conditioning status.

The CAD/CAM milled groups showed the highest flexural strength values, with Group VII (CAD/CAM Non-Aged) recording the highest mean of 149.43 ± 5.35 MPa, followed by Group VIII (CAD/CAM Aged) at 140.28 ± 5.42 MPa. Among the 3D-printed groups, the 0° (Horizontal) Non-Aged group (Group I) achieved the highest mean flexural strength at 101.14 ± 4.80 MPa, while the 90° (Vertical) Aged group (Group VI) recorded the lowest value at 70.35 ± 8.18 MPa (Table 2 and Figure 8).

Two-way ART ANOVA revealed highly significant main effects for both print orientation (*p* < 0.001, η^2^p = 0.9512) and conditioning/aging (*p* < 0.001, η^2^p = 0.5052), both with large effect sizes (Table 3). The interaction effect between Orientation and Conditioning was not statistically significant (*p* = 0.203, η^2^p = 0.0298), indicating that the aging effect was consistent across all orientation groups (Table 2).

Post hoc pairwise comparisons using Tukey’s HSD confirmed that all four orientation groups differed significantly from one another (*p* < 0.001), as shown in Table 4. The detailed Orientation × Conditioning interaction analysis showed that there is a significant difference between the different orientations with the same conditions except for the non-aged 45° and 90° (*p* = 0.401), suggesting comparable flexural performance under those specific conditions (Table 5).

### 3.2. Fatigue Resistance

**Figure 9:**
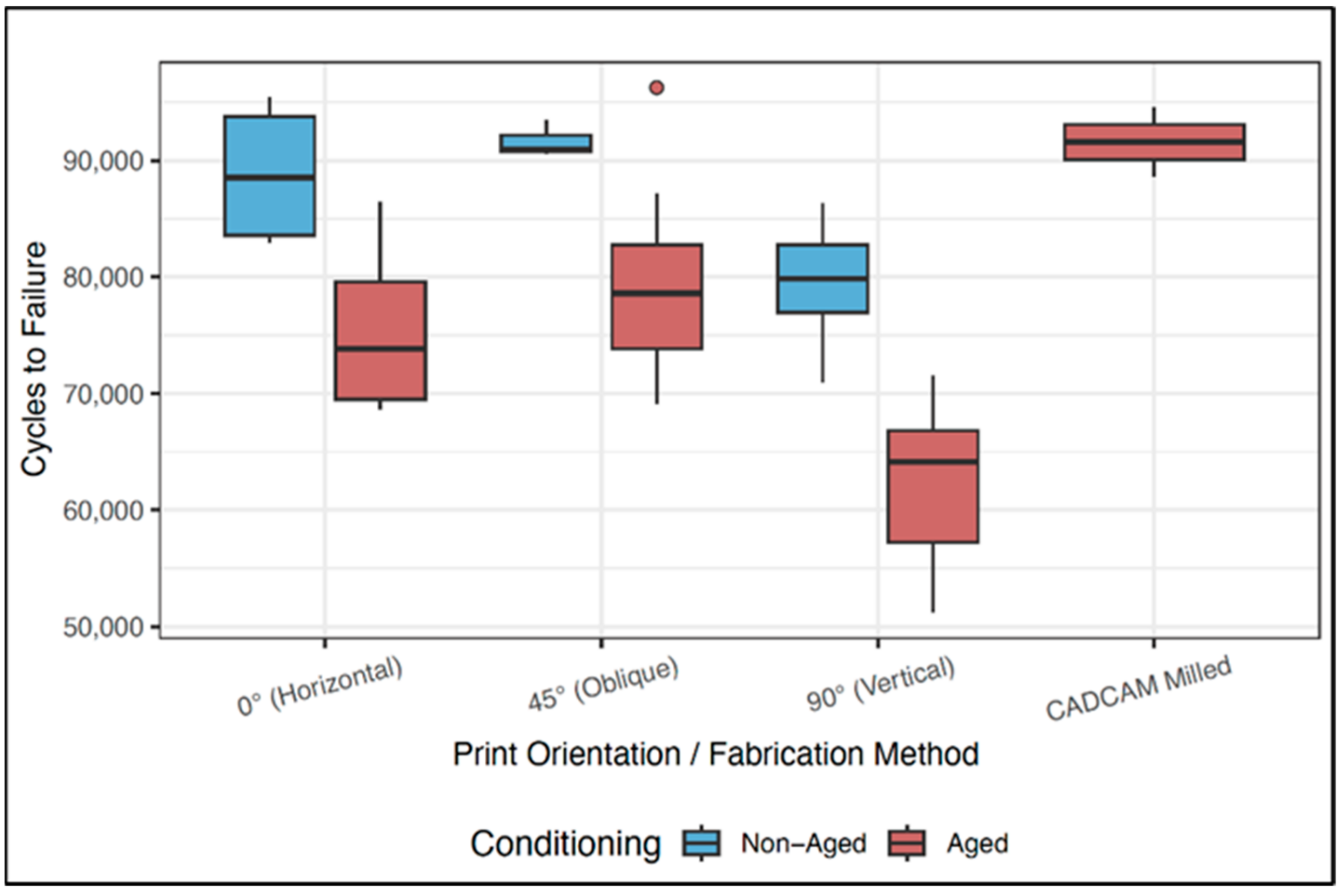
Box plot of fatigue resistance (cycles to failure) among fractured specimens across all experimental groups. Blue = Non-Aged; Orange = Aged. Runout specimens and Group VII (CADCAM Non-Aged, 0 fractures) are excluded.

CAD/CAM Non-Aged group (Group VII) showed the highest fatigue performance, with a 0% fracture rate and all 20 specimens reaching the runout threshold of 100,000 cycles. In contrast, the 90° (Vertical) Aged group (Group VI) showed the worst fatigue behavior, with a 70% fracture rate and a mean of 73,887 ± 18,459 cycles. Aging consistently increased fracture rates across all orientation groups. For example, the 0° orientation fracture rate rose from 20% (Non-Aged) to 45% (Aged), and the 90° orientation rose from 35% to 70% following artificial aging (Table 6 and Figure 9).

Because the fatigue data were not normally distributed, an ART ANOVA was used as a non-parametric alternative. The analysis showed a statistically significant effect for both Orientation (*p* < 0.001, η^2^p = 0.252) and Conditioning (*p* < 0.001, η^2^p = 0.242), as well as a significant Orientation × Conditioning interaction (F = *p* < 0.001, η^2^p = 0.163), all with large effect sizes (Table 7).

Post hoc pairwise comparisons among orientation groups (Table 8) showed that the 90° (Vertical) orientation was significantly inferior to both the 0° orientation (*p* = 0.003) and the CAD/CAM group (*p* < 0.001). No significant difference was found between the 0° and 45° groups (*p* = 0.682), nor between the 45° and CAD/CAM groups (*p* = 0.089). The Orientation × Conditioning interaction analysis (Table 9) identified the 90° Aged group as significantly inferior to all other groups (*p* ≤ 0.003), including the 90° Non-Aged group (*p* = 0.003), which highlighted the susceptibility of the vertically printed specimens to artificial aging. All CAD/CAM comparisons with non-aged 3D-printed groups were non-significant, with the exception of the 90° Aged group.

As data are not normally distributed based on Shapiro–Wilk’s normality test (*p* < 0.001) and Levene’s homogeneity test (*p* < 0.001), an Aligned Rank Transform (ART) ANOVA was performed as a non-parametric alternative to two-way ANOVA. The analysis revealed statistical significance of main effects for both Orientation and Conditioning, as well as a significant interaction effect with a large effect size (Table 8).

### 3.3. Fractography

**Figure 10:**
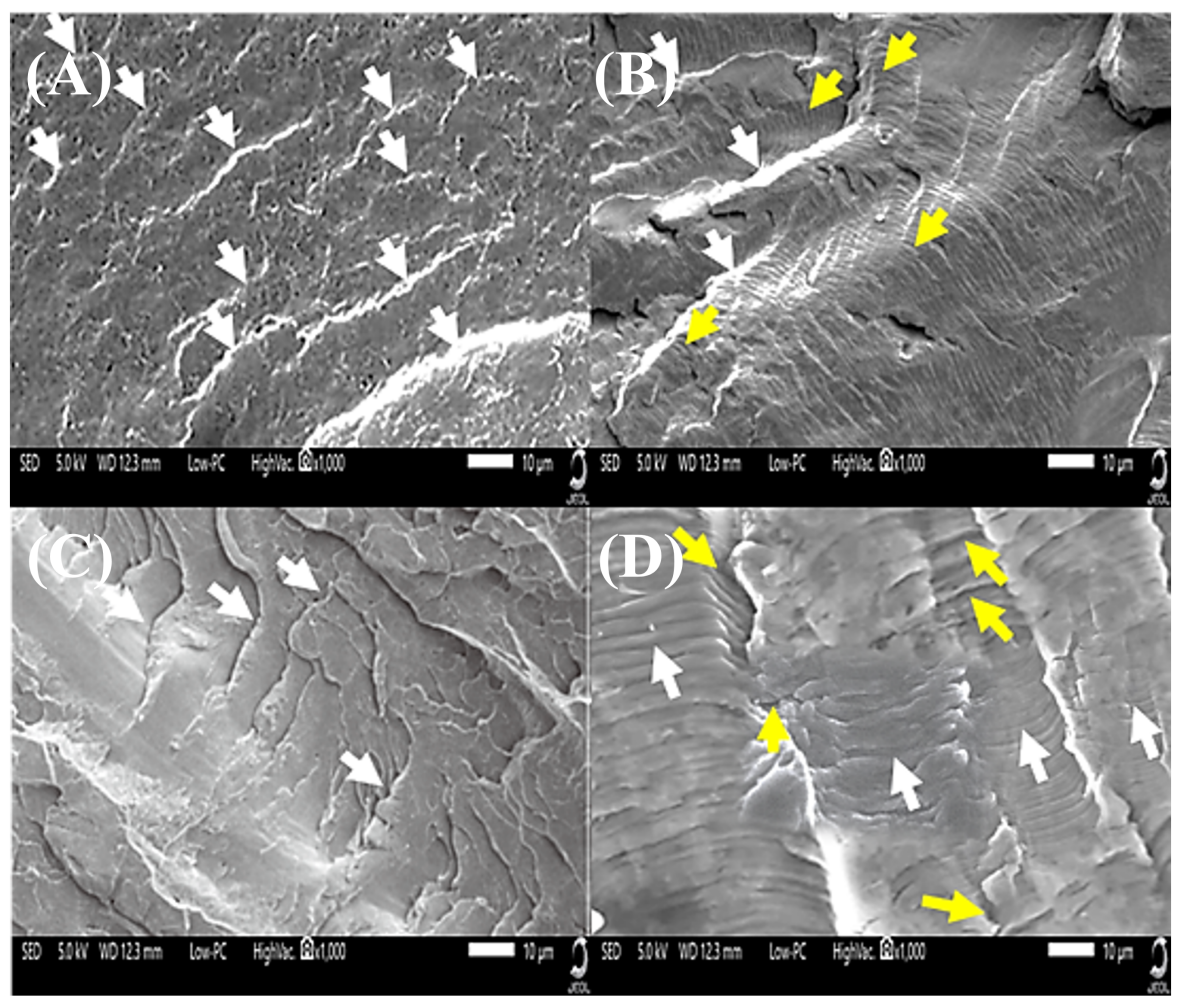
SEM of fractured surfaces of group I, (**A**) Trans-layer fracture (stair-step, white arrows), (**B**) Striations (yellow arrows) cutting across layers (white arrows). SEM of fractured surfaces of group II, (**C**) Plasticized trans-layer fracture (white arrows), (**D**) Irregular striations (white arrows), secondary cracks (yellow arrows).

The SEM features of the fractured samples’ surfaces under flexural strength fatigue resistance testing were as follows:

Group I: Horizontally printed maxillary dentures:

• SEM features of flexural strength test samples: The fracture surfaces showed characteristic “stair-step” or corrugated appearance as the crack path deviated while cutting across the horizontal layers. Sometimes, crack origin beginning at the surface was seen, followed by a rough region indicating a high-energy, sudden, catastrophic failure. Also, isolated inherent microporosities, voids from the printing process, were spotted (Figure 10A).
• SEM features of fatigue resistance test broken samples: The surfaces displayed distinct fatigue striations (arrest lines) indicating the slow, progressive growth of the crack over time. The crack path traversed the print layers, but with localized micro-cracking where the cyclic load repeatedly stressed the weak bonds between the resin layers before final fast fracture (Figure 10B).

Group II: Horizontally printed maxillary dentures subjected to thermocycling and chewing simulation:

• SEM features of flexural strength test samples: Compared to Group I, the fracture edges appeared less “crisp” and more rounded due to resin plasticization. The layers showed signs of interlayer debonding near the fracture site, as thermocycling weakens the bond between consecutive print layers (Figure 10C).
• SEM features of fatigue resistance test broken samples: The fatigue striations were present but highly irregular and “smeared” due to the softened matrix. Prominent secondary cracks branched off the main fracture line, with increased surface roughness or pitting where unreacted monomers were washed out during aging (Figure 10D).

**Figure 11:**
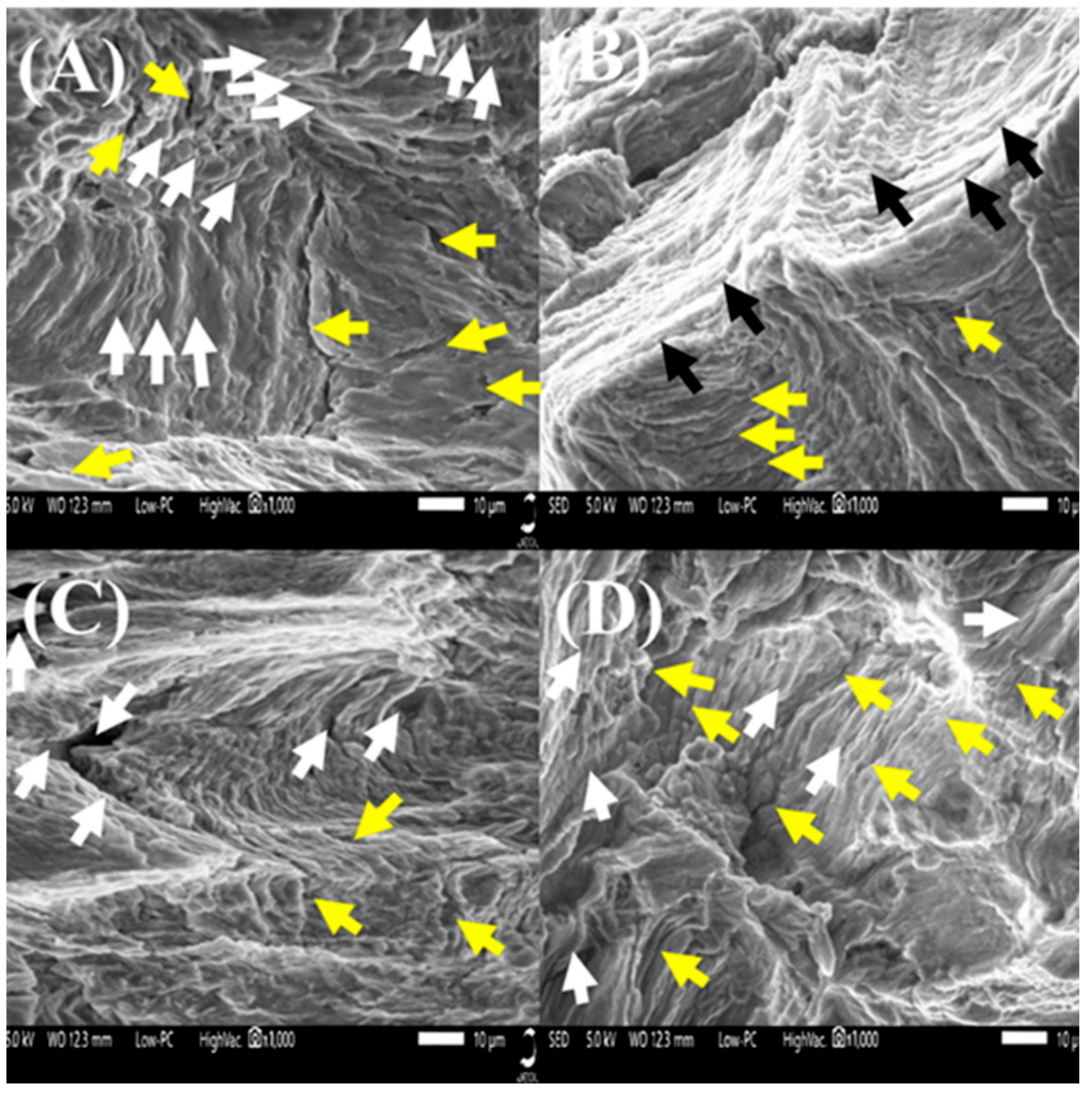
SEM of fractured surfaces of group III, (**A**) staggered, zig-zag pattern (white arrows), and short segments of interlaminar cleavage (yellow arrows). (**B**) fatigue striations (black arrows), and localized areas of structural unzipping, shear-like (yellow arrows). SEM of fractured surfaces of group IV, (**C**) prominent interlaminar gaps (white arrows), and plasticized fractured edges (yellow arrows). (**D**) Striations of the degraded surface (white arrows) and microcracking surface (yellow arrows).

Group III: Obliquely Printed Maxillary Dentures:

• SEM features of flexural strength test samples: The fracture surfaces displayed a mixed-mode failure characterized by a staggered, zig-zag pattern (white arrows). The crack propagation path deflected diagonally, transitioning between trans-layer fractures, cutting through the resin, with short segments of interlaminar cleavage traveling along the 45° layer interfaces (yellow arrows) (Figure 11A).
• SEM features of fatigue resistance test broken samples: Under cyclic loading, fatigue striations appeared angled relative to the primary stress axis (black arrows). Localized areas of “structural unzipping” (shear-like sliding) along the 45° layer boundaries were visible prior to the fast fracture zone (yellow arrows) (Figure 11B).

Group IV: Obliquely printed maxillary dentures subjected to thermocycling and chewing simulation:

• SEM features of flexural strength test samples: The SEM showed more prominent interlaminar gaps compared to the unaged Group III (white arrows). The fractured edges looked more plasticized (yellow arrows) (Figure 11C).
• SEM features of fatigue resistance test broken samples: The SEM showed pronounced striations of the degraded surface under cyclic loading (white arrows). The mechanical washout of unreacted monomers during aging resulted in more surface micro-cracks (yellow arrows) (Figure 11D).

**Figure 12:**
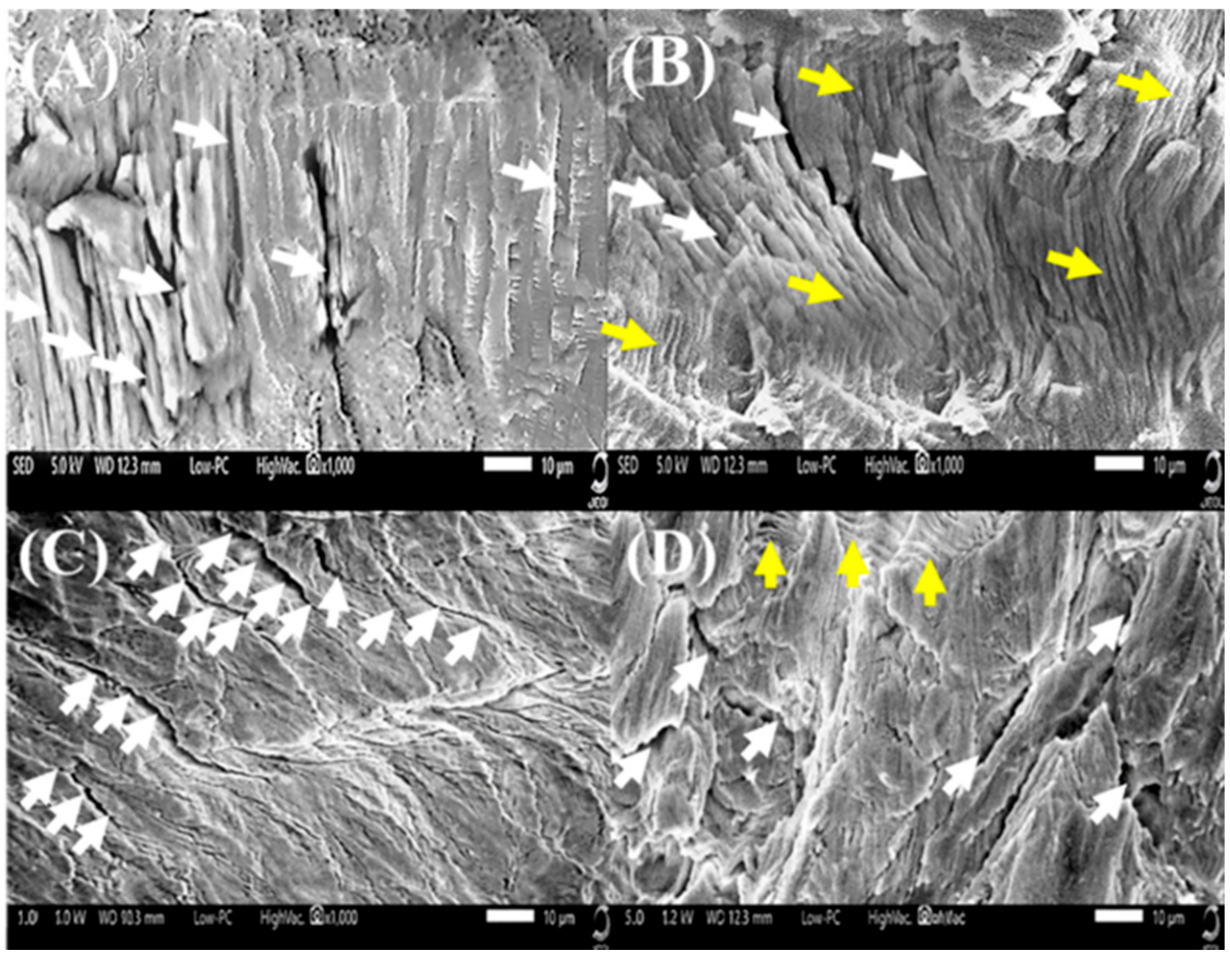
SEM of fractured surfaces of group V, (**A**) Interlaminar cleavage (white arrows), (**B**) Interlaminar cleavage (white arrows), and interfacial fatigue striations (yellow arrows). SEM of fractured surfaces of group VI, (**C**) Severe adhesive failure (gaps), (**D**) Widespread structural unzipping (white arrows), and interfacial fatigue striations (yellow arrows). White arrows shows severe adhesive failure.

Group V: Vertically Printed Maxillary Dentures:

• SEM features of flexural strength test samples: The hallmark of this subgroup was the adhesive failure (delamination). The SEM showed a relatively smooth, “cleavage-like” fracture surface because the static load simply split or peeled the vertical layers apart along their interfaces. Hackle lines were minimal, indicating a low-energy failure (Figure 12A).
• SEM features of fatigue resistance test broken samples: Pronounced layer separation. Under cyclic loading, the continuous flexing caused the vertical layers to “unzip.” And fatigue striations were strictly confined within the adhesive interface between layers, rather than across the bulk material (Figure 12B).

Group VI: Vertically printed maxillary dentures subjected to thermocycling and chewing simulation:

• SEM features of flexural strength test samples: The SEM showed clear, wide fissures between the vertical layers, demonstrating that the thermocycling completely degraded the interlayer adhesion. The fracture surface will look like a separated deck of cards (Figure 12C).
• SEM features of fatigue resistance test broken samples: The SEM showed multiple points of crack initiation. The matrix also showed signs of swelling, and the separation of layers was profound, with deep, branching secondary cracks traveling vertically down the denture base (Figure 12D).

**Figure 13:**
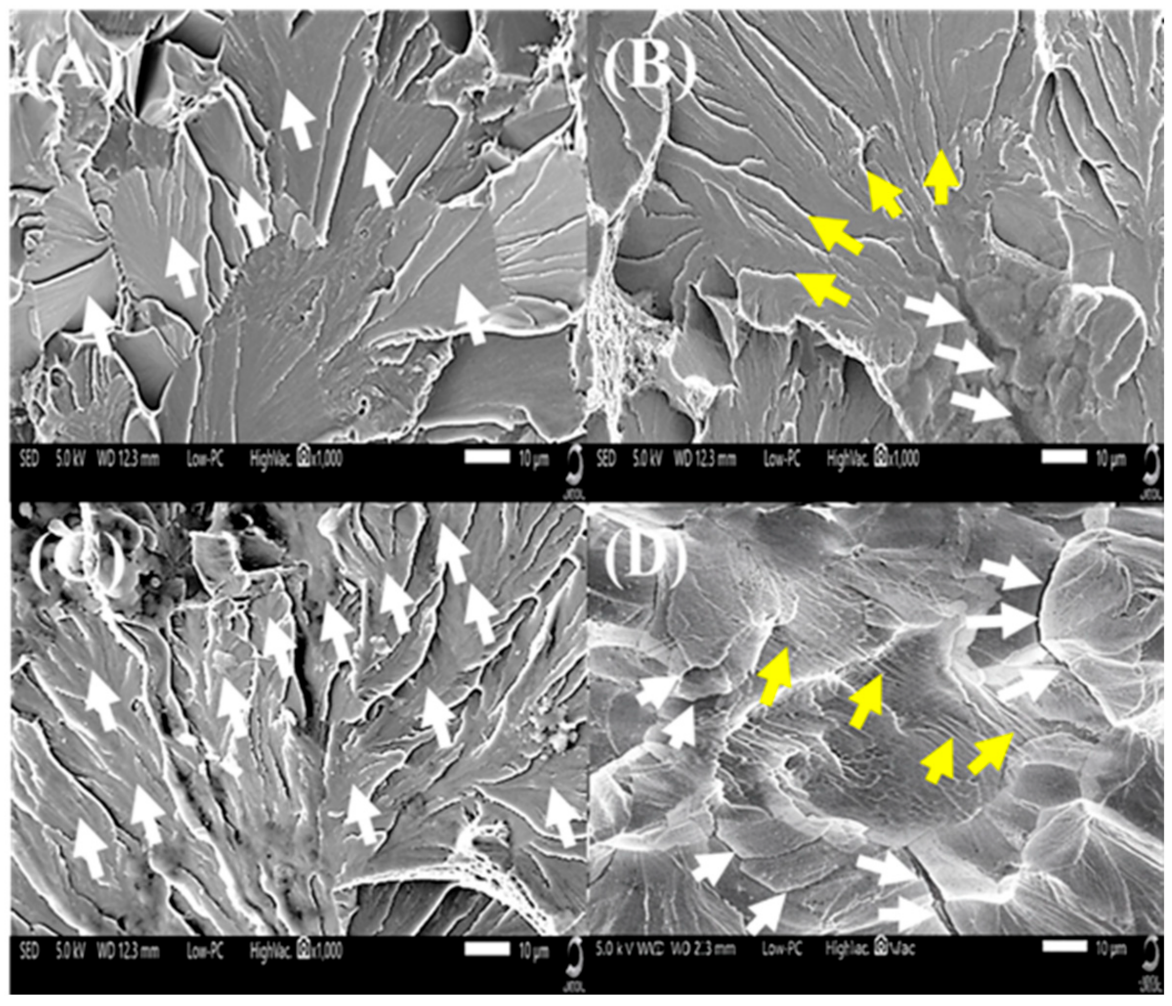
SEM of fractured surfaces of group VII, (**A**) Classic Mirror-Mist-Hackle (white arrows), (**B**) Origin of fracture (white arrows), and uniform, clear fan-like striations (yellow arrows). SEM of fractured surfaces of group VIII, (**C**) Smaller mirror zone, fast fracture, (white arrows), (**D**) Surface micro-cracking (white arrows), and striations (yellow arrows).

Group VII: CAD/CAM Milled Maxillary Dentures:

• SEM features of flexural strength test samples: The SEM revealed homogeneous brittle and semi-brittle polymer fracture, no layer lines and highly dense surfaces with virtually no porosity. The fracture sites featured clear “mirror” zones radiating outward to a “mist” and finally a rough “hackle” zone (Figure 13A).
• SEM features of fatigue resistance test broken samples: The SEM revealed clear, uniform fatigue striations radiating in a fan-like pattern from a single initiation point. The surrounding matrix appeared highly intact and dense, with very few secondary cracks compared to the printed groups (Figure 13B).

Group VIII: CAD/CAM milled maxillary dentures subjected to thermocycling and chewing simulation:

• SEM features of flexural strength test samples: The SEM revealed similar morphology to group V, but the “mirror” zones were smaller, indicating that less energy was required to initiate fast fracture due to aging. Slight surface degradation and microscopic shallow pitting were visible due to water sorption (Figure 13C).
• SEM features of fatigue resistance test broken samples: The SEM showed clear fatigue striations but were accompanied by surface micro-cracking and roughening caused by the chewing simulation’s localized stresses. The crack propagation zones showed slightly more texturing and matrix yielding compared to the unaged control (Figure 13D).

## 4. Discussion

The results of the present study led to the rejection of the null hypothesis. Both printing orientation and thermomechanical aging significantly influenced the mechanical behavior of 3D-printed denture bases. Furthermore, significant differences were observed between the tested printing orientations and the CAD/CAM milled control group with respect to flexural strength and fatigue resistance.

The findings of the present study confirm the inherently anisotropic nature of additively manufactured polymers. Horizontally printed specimens consistently exhibited superior flexural strength compared to oblique and vertical orientations, which aligns with previous investigations reporting improved mechanical performance when the applied load is parallel to the printed layers [9,10,11]. This behavior can be attributed to the more effective distribution of stresses along the layer interfaces, allowing the structure to rely predominantly on cohesive bulk properties rather than weaker interlayer bonds [7,9].

In contrast, vertically printed specimens demonstrated the lowest flexural strength, which can be explained by the orientation of the load relative to the layer interfaces. When stress is applied perpendicular to the layers, failure tends to initiate and propagate along interfacial boundaries, which are inherently weaker due to incomplete polymerization and limited interlayer diffusion [10,11]. The oblique (45°) orientation produced intermediate results, reflecting a combined failure mechanism involving both cohesive fracture and interfacial debonding, as also reported in the previous literature [7,12,13,14,15,16,17].

Thermomechanical aging had a significant detrimental effect on flexural strength across all groups, which is in agreement with previous studies highlighting the role of water sorption and thermal cycling in degrading polymer networks [2,20,37,40,43]. The absence of a significant interaction between print orientation and aging suggests that hydrolytic degradation and matrix plasticization occur relatively uniformly regardless of layer orientation. This indicates that, under static loading conditions, the dominant mechanism of degradation is related to bulk material softening rather than interfacial failure [2,6,24,25,26].

However, a markedly different trend was observed under cyclic loading conditions. Fatigue resistance was significantly affected not only by print orientation and aging independently, but also by their interaction. This finding underscores the importance of evaluating dental materials under dynamic loading conditions that more closely simulate clinical function. Vertically printed aged specimens exhibited the highest fracture rate, reaching 70%, which reflects a pronounced susceptibility to fatigue-induced interlaminar failure. This behavior can be explained by the repetitive opening and propagation of microcracks along weakened interlayer interfaces, a phenomenon previously described in studies investigating fatigue behavior of layered polymeric structures [6,10,22,23].

Horizontally printed specimens demonstrated greater resistance to fatigue failure, likely because crack propagation was forced to traverse across multiple layers rather than propagate along them. This results in higher energy dissipation during crack growth and delays catastrophic failure. Similar observations have been reported in studies evaluating the fatigue performance of additively manufactured dental resins, where favorable layer alignment significantly improved resistance to cyclic loading [9,19].

The oblique orientation again demonstrated intermediate behavior. While it reduced the likelihood of immediate delamination compared to vertical builds, it still allowed progressive crack propagation along inclined interfaces under cyclic loading. This mixed-mode failure pattern highlights the complexity of stress distribution in obliquely printed structures and supports previous findings suggesting that 45° orientation represents a compromise rather than an optimal solution [7,9,12].

Fractographic analysis provided further insight into the underlying failure mechanisms. Horizontally printed specimens exhibited trans-layer fracture patterns with distinct fatigue striations crossing the layers, indicating cohesive failure within the material. In contrast, vertically printed specimens showed clear evidence of interlaminar delamination, characterized by smooth, cleavage-like surfaces and minimal energy absorption prior to failure. These observations are consistent with previous SEM-based studies that identified interfacial separation as the dominant failure mode in vertically printed polymers [10,11]. The oblique groups displayed a combination of these features, with crack deflection and zig-zag propagation paths reflecting alternating cohesive and adhesive failure modes.

Despite optimizing print orientation, all 3D-printed groups remained inferior to CAD/CAM milled specimens in both flexural strength and fatigue resistance. This finding is well supported by the literature, which consistently reports superior mechanical properties for milled PMMA due to its highly polymerized, dense, and homogeneous structure [1,4,5]. The absence of interlayer defects and the industrially controlled polymerization process contribute to improved structural integrity and long-term durability [3,6].

The superior performance of CAD/CAM milled dentures was particularly evident under fatigue testing, where non-aged specimens demonstrated complete survival without fracture. Even after aging, their performance remained significantly higher than that of all printed groups. These findings reinforce the position of milled denture bases as the current gold standard for definitive prosthetic applications, especially in patients with high functional demands [4,5,19].

An important clinical observation from this study is the consistent occurrence of fractures along the palatal midline. This region is known to be a critical stress concentration area in maxillary complete dentures, particularly under flexural loading conditions [18]. While horizontal printing improved resistance to crack propagation in this region, the orientation-dependent nature of failure suggests that clinicians must carefully consider build direction when designing 3D-printed prostheses.

From a clinical perspective, horizontal printing should be considered the preferred orientation whenever feasible, as it provides the best balance between strength and fatigue resistance. Oblique printing may be used when geometric or manufacturing constraints prevent horizontal placement, but with the understanding that it offers only moderate performance. Vertical printing, on the other hand, should be avoided for definitive prostheses subjected to long-term functional loading due to its high susceptibility to interlaminar failure.

Although 3D printing is efficient in terms of customization and material utilization, its mechanical limitations remain a concern. For definitive prosthetic patients with parafunctional habits or high occlusal loads, CAD/CAM milled denture bases continue to provide a more reliable solution. Nevertheless, while our results clearly show CAD/CAM milled samples possess superior mechanical integrity, 3D printing remains highly feasible and advantageous due to several compelling clinical and economic factors. It significantly reduces material subtractive waste, allows for the rapid fabrication of custom provisional dentures, offers a more streamlined digital workflow, and requires a much lower initial equipment investment compared to industrial milling machines. Furthermore, the horizontally printed dentures demonstrated sufficient structural strength that may be clinically acceptable for short-to-medium-term use, interim, diagnostic, and low-load cases; however, they are not yet a full replacement for CAD/CAM in high-load situations [38,39,40,42,43].

The current work suffered several limitations. First, the absence of typical oral cavity conditions may influence the long-term degradation of polymer chains. Second, the fatigue test utilized a constant cyclic load at a specific frequency. In a clinical setting, masticatory forces are multi-directional, non-linear, and vary significantly between patients based on neuromuscular coordination and bolus consistency. Third, only one specific commercial brand of 3D-printing resin and CAD/CAM blocks was tested. Given the rapid evolution of polymer chemistry, results may vary with different printing or different post-curing units, which are known to significantly affect the degree of conversion; therefore, the severe anisotropic degradation observed, particularly the catastrophic interlaminar delamination in the vertically printed groups, may be intimately tied to the photopolymerization dynamics of this specific material. These findings cannot be universally extrapolated to all 3D-printed dentures, especially emerging composite resins incorporating ceramic or glass fillers [16,21]. Fourth, specimens were tested shortly after fabrication. Clinical dentures undergo water sorption and “aging” over years of use, which typically reduces the flexural strength and increases the susceptibility of the midline to fatigue failure over time. Fifth, the fatigue testing protocol utilized a uniaxial load applied solely to the center of the palate. In true clinical function, masticatory forces are primarily localized over the posterior alveolar ridges, generating complex, multi-directional bending moments across the midline. While central palatal loading oversimplifies the intraoral stress distribution, it serves as a standardized, reproducible ‘worst-case scenario’ designed specifically to induce midline flexural fatigue. Because midline fracture is the most common mode of catastrophic failure in maxillary complete dentures, this protocol is highly effective for comparative material testing, though it suggests our reported cycles-to-failure may be lower than what would be observed under distributed clinical loading. Finally, only flexural strength and fatigue resistance were evaluated; in addition to the fact that a single resin material was evaluated, the findings of this study may call for future research to confirm their validity.

In conclusion, careful selection of print orientation is essential when fabricating 3D-printed dentures. Horizontal orientation should be prioritized whenever possible to enhance longevity, while vertical printing should be avoided in cases where long-term mechanical performance is critical. Although additive manufacturing offers clear benefits in efficiency and customization, within the limitations of this laboratory study and the specific resin materials tested, CAD/CAM milled dentures demonstrated superior durability under simulated aging conditions, suggesting they may be more suitable for definitive, long-term applications compared to the evaluated 3D-printed groups.

## 5. Conclusions

Within the limitations of this in vitro study, both printing orientation and thermomechanical aging significantly influenced the mechanical performance of 3D-printed denture bases. Horizontally printed dentures demonstrated the highest flexural strength and fatigue resistance among the printed groups, whereas vertically printed dentures showed the poorest performance, particularly after aging. Although optimization of print orientation improved mechanical behavior, all 3D-printed groups remained inferior to CAD/CAM milled denture bases, which exhibited the greatest strength and durability under both static and cyclic loading conditions.

## Figures and Tables

**Table 1:** Summary of experimental groups.

Group	Fabrication Method/Printing Direction	Aging Condition	Flexural Strength Testing Samples	Fatigue Testing Samples
I	0° (Horizontal)	Non-Aged	20	20
II	0° (Horizontal)	Aged	20	20
III	45° (Oblique)	Non-Aged	20	20
IV	45° (Oblique)	Aged	20	20
V	90° (Vertical)	Non-Aged	20	20
VI	90° (Vertical)	Aged	20	20
VII	CADCAM	Non-Aged	20	20
VIII	CADCAM	Aged	20	20

**Table 2:** Descriptive statistics of flexural strength results across all experimental groups.

Group	Method/Aging	Mean ± SD (Mpa)	Min	Max
Group I (N = 20)	0° (Horizontal) Non-Aged	101.14 ± 4.80	92.43	109.90
Group II (N = 20)	0° (Horizontal) Aged	90.40 ± 5.81	80.24	103.11
Group III (N = 20)	45° (Oblique) Non-Aged	93.84 ± 4.93	83.42	100.34
Group IV (N = 20)	45° (Oblique) Aged	80.78 ± 7.78	62.66	91.95
Group V (N = 20)	90° (Vertical) Non-Aged	84.83 ± 4.84	74.76	95.35
Group VI (N = 20)	90° (Vertical) Aged	70.35 ± 8.18	54.65	89.71
Group VII (N = 20)	CADCAM Non-Aged	149.43 ± 5.35	142.25	160.95
Group VIII (N = 20)	CADCAM Aged	140.28 ± 5.42	130.36	151.19

N is the number of samples in each group.

**Table 3:** Two-Way ANOVA showing the effects of Orientation and Conditioning, and their interaction on flexural strength.

Source	F	*p*	Partial η^2^p
Orientation	988.13	<0.001 *	0.9512
Conditioning	155.20	<0.001 *	0.5052
Orientation × Conditioning	1.55	0.203	0.0298

F: Two-way ANOVA, Partial η^2^: for effect size, * significant at *p* < 0.05.

**Table 4:** Pairwise comparisons of flexural strength among print orientation groups.

	45° (Oblique)	90° (Vertical)	CADCAM Milled
0° (Horizontal)	<0.001 *	<0.001 *	<0.001 *
45° (Oblique)		<0.001 *	<0.001 *
90° (Vertical)			<0.001 *

*p*: for Tukey post Hoc, * significant at *p* < 0.05.

**Table 5:** Pairwise comparisons of flexural strength among all Orientation × Conditioning combinations.

	0° Aged	45° Non-Aged	45° Aged	90° Non-Aged	90° Aged	CADCAM Non-Aged	CADCAM Aged
0° Non-Aged	<0.001 *	0.004 *	<0.001 *	<0.001 *	<0.001 *	<0.001 *	<0.001 *
0° Aged		0.618	<0.001 *	0.075	<0.001 *	<0.001 *	<0.001 *
45° Non-Aged			<0.001 *	0.401	<0.001 *	<0.001 *	<0.001 *
45° Aged				<0.001 *	<0.001 *	<0.001 *	<0.001 *
90° Non-Aged					<0.001 *	<0.001 *	<0.001 *
90° Aged						<0.001 *	<0.001 *
CADCAM Non-Aged							<0.001 *

*p*: for Tukey post Hoc, * significant at *p* < 0.05.

**Table 6:** Descriptive statistics of fatigue resistance results across all experimental groups.

Group	Method/Aging	Fractured	Runout	Fracture Rate	All (Mean ± SD)	Fractured Only (Mean ± SD)
Group I (N = 20)	0° (Horizontal) Non-Aged	4	16	20%	97,764 ± 5244	88,820 ± 6389
Group II (N = 20)	0° (Horizontal) Aged	9	11	45%	88,865 ± 13,236	75,256 ± 6101
Group III (N = 20)	45° (Oblique) Non-Aged	3	17	15%	98,748 ± 3100	91,653 ± 1565
Group IV (N = 20)	45° (Oblique) Aged	8	12	40%	91,828 ± 11,607	79,571 ± 8917
Group V (N = 20)	90° (Vertical) Non-Aged	7	13	35%	92,830 ± 10,451	79,513 ± 5252
Group VI (N = 20)	90° (Vertical) Aged	14	6	70%	73,887 ± 18,459	62,696 ± 6958
Group VII (N = 20)	CADCAM Non-Aged	0	20	0%	100,000 ± 0	—
Group VIII (N = 20)	CADCAM Aged	2	18	10%	99,158 ± 2767	91,580 ± 4228

**Table 7:** ART ANOVA showing the effects of Orientation and Conditioning, and their interaction on fatigue resistance (cycles to failure).

Source	F	*p*	Partial η^2^
Orientation	17.07	<0.001 ***	0.252
Conditioning	48.54	<0.001 ***	0.242
Orientation × Conditioning	9.85	<0.001 ***	0.163

F: for ART ANOVA, Partial η^2^: for effect size, *** denotes significant difference at *p* < 0.001.

**Table 8:** Pairwise comparisons of fatigue resistance among print orientation groups.

	45° (Oblique)	90° (Vertical)	CADCAM
0° (Horizontal)	0.682	0.003 *	0.004 *
45° (Oblique)		<0.001 *	0.089
90° (Vertical)			<0.001 *

*p*: for Tukey post Hoc, * significant at *p* < 0.05.

**Table 9:** Pairwise comparisons of fatigue resistance among all Orientation × Conditioning combinations.

	0° Aged	45° Non-Aged	45° Aged	90° Non-Aged	90° Aged	CADCAM Non-Aged	CADCAM Aged
0° Non-Aged	0.591	1.000	1.000	1.000	<0.001 *	1.000	1.000
0° Aged		0.222	1.000	1.000	0.092	0.012 *	0.089
45° Non-Aged			1.000	1.000	<0.001 *	1.000	1.000
45° Aged				1.000	0.012 *	1.000	0.526
90° Non-Aged					0.003 *	1.000	1.000
90° Aged						<0.001 *	<0.001 *
CADCAM Non-Aged							1.000

*p*: Bonferroni adjustment for 28 comparisons. * = significant at *p* < 0.05.

## Data Availability

The original contributions presented in this study are included in the article. Further inquiries can be directed to the corresponding author.

## Abbreviations

CAD/CAM	Computer aided design/computer aided manufacturing
SEM	Scanning electron microscope
PMMA	polymethyl methacrylate
CAD file	Computer-aided design file
kN	Kilo newton
MPa	Mega pascal
N	Newton
Hz	Hertz
Au	gold
kV	Kilo volt
ART	Aligned Rank Transform
η^2^p	Partial Eta Squared
Tukey’s HSD	Tukey’s Honestly Significant Difference test
Mean ± SD	mean ± standard deviation
